# Synthesis and Photophysical Characterization of Fluorescent Naphtho[2,3-*d*]thiazole-4,9-Diones and Their Antimicrobial Activity against *Staphylococcus* Strains

**DOI:** 10.3390/molecules29122777

**Published:** 2024-06-11

**Authors:** Masayori Hagimori, Fumiko Hara, Naoko Mizuyama, Shinya Takada, Saki Hayashi, Tamami Haraguchi, Yoshiro Hatanaka, Toshihiro Nagao, Shigemitsu Tanaka, Miki Yoshii, Miyako Yoshida

**Affiliations:** 1 Department of Analitical Chemistry, Faculty of Pharmaceutical Sciences, Mukogawa Women’s University, 11-68 Koshien 9-Bancho, Nishinomiya City 663-8179, Hyogo, Japan; fhara@mukogawa-u.ac.jp (F.H.); takada_shinya_x@mukogawa-u.ac.jp (S.T.); 2 Division of Medical Innovation, Translational Research Center for Medical Innovation, 1-5-4 Minatojima-minamimachi, Chuo-ku, Kobe 650-0047, Hyogo, Japan; hagimori@fbri.org; 3 Department of Clinical Pharmaceutics, Faculty of Pharmaceutical Sciences, Mukogawa Women’s University, 11-68 Koshien 9-Bancho, Nishinomiya City 663-8179, Hyogo, Japan; hayashi_saki_x@mukogawa-u.ac.jp (S.H.); tsuchiko_tamami_x@mukogawa-u.ac.jp (T.H.); 4 Institute for Women’s Career Advancement and Gender Equality Development, Mukogawa Women’s University, 6-46 Ikebiraki, Nishinomiya City 663-8558, Hyogo, Japan; 5 Osaka Research Institute of Industrial Science and Technology, 1-6-50 Morinomiya, Joto-ku, Osaka City 536-8553, Osaka, Japan; hatanaka@orist.jp (Y.H.); nagao@orist.jp (T.N.); s-tanaka@orist.jp (S.T.); yoshii@orist.jp (M.Y.)

**Keywords:** naphtho[2,3-*d*]thiazole-4,9-dione, fluorescence, bathochromic shift, antimicrobial activity, *Staphylococcus*

## Abstract

The chemical reaction of 2-(methylsulfinyl)naphtho[2,3-*d*]thiazole-4,9-dione (**3**) using different amines, including benzylamine (**4a**), morpholine (**4b**), thiomorpholine (**4c**), piperidine (**4d**), and 4-methylpiperazine (**4e**), produced corresponding new tricyclic naphtho[2,3-*d*]thiazole–4,9–dione compounds (**5a**–**e**) in moderate-to-good yields. The photophysical properties and antimicrobial activities of these compounds (**5a**–**e**) were then characterized. Owing to the extended π-conjugated system of naphtho[2,3-*d*]thiazole–4,9–dione skeleton and substituent effect, **5a**–**e** showed fluorescence both in solution and in the solid state. The introduction of nitrogen-containing heterocycles at position 2 of the thiazole ring on naphtho[2,3-*d*]thiazole-4,9-dione led to large bathochromic shifts in solution, and **5b**–**e** exhibited orange-red fluorescence with emission maxima of over 600 nm in highly polar solvents. *Staphylococcus aureus* (*S. aureus*) is a highly pathogenic bacterium, and infection with its antimicrobial-resistant pathogen methicillin-resistant *S. aureus* (MRSA) results in serious clinical problems. In this study, we also investigated the antimicrobial activities of **5a**–**e** against *S. aureus*, MRSA, and *S. epidermidis*. Compounds **5c** with thiomorpholine group and **5e** with 4-methylpiperazine group showed potent antimicrobial activity against these bacteria. These results will lead to the development of new fluorescent dyes with antimicrobial activity in the future.

## 1. Introduction

Fluorescence is an attractive tool used in a wide range of scientific fields, from molecular biology and biochemistry to materials science and medical diagnostics [1,2,3,4]. The unique ability of fluorescence to emit light following excitation has enabled researchers to elucidate complex biological processes, develop advanced sensing technologies, and design new materials for diverse applications [1,2,3,4]. Various fluorescent substances based on small organic molecules, proteins, and quantum dots have been reported; small fluorescence organic molecules based on coumarin, fluorescein, and rhodamine have easily modifiable structures and their emission wavelength, emission intensity, and photosensitivity can be optimized by introducing substituents [5,6,7]. In addition, small organic molecules often exhibit solvatochromism in their absorption and emission spectra, which is useful in the design of fluorescent probes, sensors, and optical sensing devices [8,9]. Owing to these properties, fluorescent small organic molecules could be applied in various fields; the development of small-molecule-based fluorescent substances has received increased interest in recent years.

**Figure 1:**
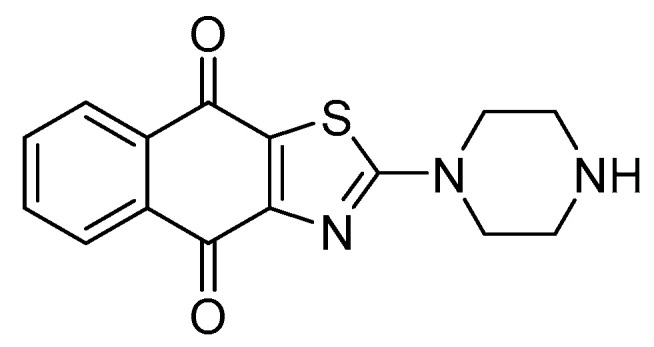
Chemical structure of PNT.

Naphtho[2,3-*d*]thiazole-4,9-dione compound, comprising 1,4-naphthoquinone skeleton and a thiazole ring, is frequently found in pharmaceutical compounds with anti-inflammatory, anticancer, and antibacterial activities [10,11,12,13]. Naphthoquinone structure has been reported to affect these activities by producing reactive oxygen species such as singlet oxygen, superoxide, and oxygen free radicals and regulating redox signaling in biological systems [14,15]. Thiazole structure is an aromatic five-membered ring, which exhibits antimicrobial activity when it is used alone or in combination with other heterocycles, such as aztreonam, abafungin, and isavuconazole [14,15]. Aztreonam, the first synthetic monobactam antibiotic, showed excellent antimicrobial activity against Gram-negative, aerobic bacteria, while abafungin and isavuconazole are cephalosporin antibiotics and have been reported to exhibit antifungal activity. Therefore, derivatives based on the naphtho[2,3-*d*]thiazole-4,9-dione skeleton could exhibit these bioactive properties. Naphtho[2,3-*d*]thiazole-4,9-dione compound (NSC631527) with (2-chlorophenyl)amino group was identified by high-throughput screening to have cytotoxic properties in the fission yeast *Schizosaccharomyces pombe* [16]. A structure–activity–relationship study revealed that substitution at position 2 of the thiazole ring on naphtho[2,3-*d*]thiazole-4,9-dione inhibited proliferation activities of cancers, including MDA-MB-231 human breast cancer cells, HeLa cervical cancer cells, and MKN-45 human gastric cancer cells; docking simulations showed that the prepared compounds may attach to the hDNA TopoIIβ binding pocket [17]. Current research on the production of biologically active naphtho[2,3-*d*]thiazole-4,9-diones is focused on the introduction of various substituents and the development of novel synthetic methodologies [11,18]. Numerous studies have explored the fluorescence characteristics of naphthoquinones [19,20,21]; however, research reports specifically pertaining to naphtho[2,3-*d*]thiazole-4,9-diones are noticeably scarce despite their substantial extension of the π-conjugated system. Recently, naphtho[2,3-*d*]thiazole-4,9-dione, having a piperazino group at position 2 of the thiazole ring (PNT) (Figure 1), was reported to have antimicrobial activity against the staphylococcal strains, as well as fluorescence around 440–490 nm and a large Stokes shift (>90 nm) [22]. The large Stokes shift allows fluorescence to be measured without considerable effect from excitation light, making them particularly useful in spectral analysis, sensing, and optical devices. However, the photophysical properties of PNT have not been fully investigated and there have been no reports on PNT derivatives.

To address this gap in the literature, we report a method to synthesize novel naphtho[2,3-*d*]thiazole-4,9-diones (**5a**–**e**) and their photophysical properties both in solution and the solid state. Based on the findings in PNT and other derivatives, the introduction of nitrogen-containing heterocyclic groups at position 2 of the thiazole ring on naphtho[2,3-*d*]thiazole-4,9-dione caused bathochromic shifts in both the absorption and emission wavelengths of the resulting compounds. The emission maxima of **5b**–**e** showed pronounced bathochromic shifts of over 600 nm in polar solvents. The antimicrobial activity of **5a**–**e** toward *S. aureus*, methicillin-resistant *S. aureus* (MRSA), and *S. epidermidis* was also studied to evaluate their biological usefulness.

## 2. Results and Discussion

**Scheme 1:**
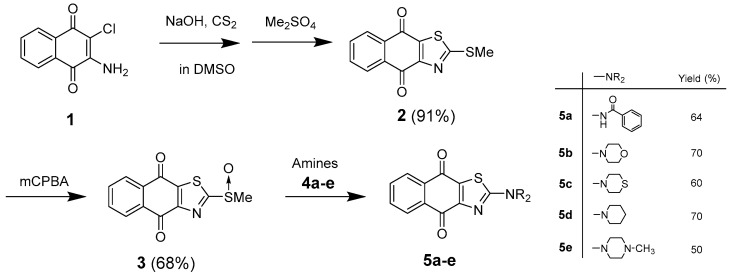
Syntheses of **5a**–**e**.

Naphtho[2,3-*d*]thiazole-4,9-dione compounds (**5a**–**e**) were prepared from 2-(methylsulfinyl)naphtho[2,3-*d*]thiazole-4,9-dione (**3**) with amines (**4a**–**e**), as shown in Scheme 1. Briefly, compound **3** was prepared in two steps using commercially available 2-amino-3-chloronaphthalene-1,4-dione (**1**) as the starting compound. The reaction of **1** with carbon disulfide in DMSO using sodium hydroxide as a base at room temperature in DMSO followed by dimethyl sulfate gave 2-(methylthio)naphtho[2,3-*d*]thiazole-4,9-dione (**2**) in 91% yield. Thereafter, the oxidation of **2** was achieved using mCPBA, and compound **3** was obtained in a 68% yield. *N*-(4,9-dioxo-4,9-dihydronaphtho[2,3-*d*]thiazol-2-yl)benzamide (**5a**), which has been reported to have antimicrobial activity against *Candida albicans*, *Aspergillus fumigatus*, *S. aureus,* and *S. epidermidis* [23,24], was easily synthesized in a one-pot synthesis from compound **3** and benzylamine (**4a**) at 100 °C for 2 h to 61% yield. Compounds **5b**–**e** bearing nitrogen-containing heterocycles at position 2 of the thiazole ring were prepared in moderate-to-good yields (50–70%) using a similar synthetic method via the reaction of **3** with heterocyclic amines including morpholine (**4b**), thiomorpholine (**4c**), piperidine (**4d**), and 4-methylpiperazine (**4e**). HPLC-UV results show that the purity of all compounds was more than 95% (Figures S1–S6).

**Figure 2:**
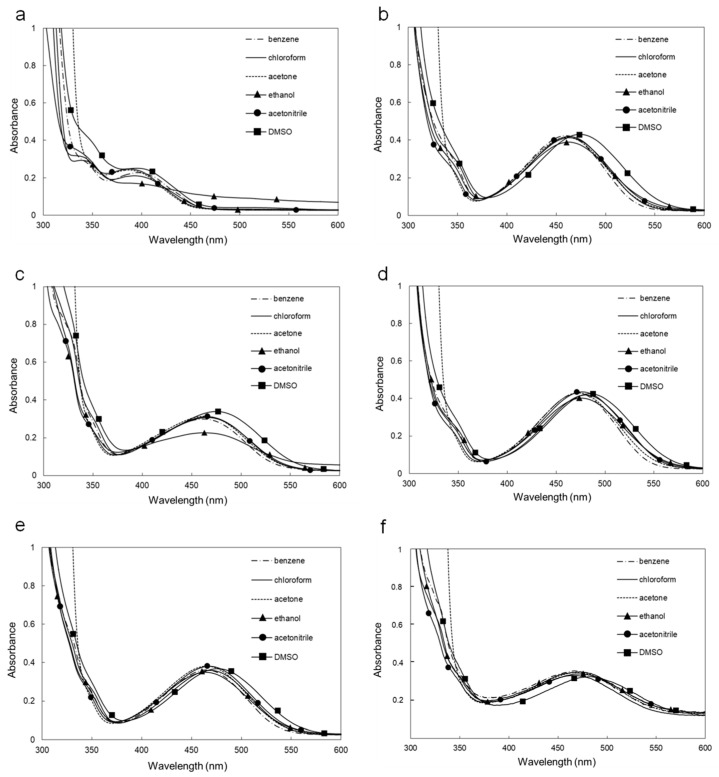
Absorption spectra of **5a**–**e** and PNT (**a**: **5a**, **b**: **5b**, **c**: **5c**, **d**: **5d**, **e**: **5e** and **f**: PNT) in benzene, chloroform, acetone, ethanol, acetonitrile, DMSO in benzene, chloroform, ethanol, acetone, acetonitrile, DMSO, (1 × 10^−4^ mol/L).

The photophysical properties (**5a**–**e**), including their maximum absorption wavelength (λ_max_), molar extinction coefficient (log ε), maximum emission wavelength (Em_max_), and fluorescence quantum yield (Φ), were measured in solvents with different polarities (Table 1 and Table 2). PNT was also measured for comparative purposes. The UV–Vis absorption spectra of **5a**–**e** and PNT in benzene, chloroform, acetone, ethanol, acetonitrile, and DMSO are shown in Figure 2. Compound **5a** exhibited absorption in the UV–Vis range in all measured solvents, irrespective of the polarity of the solvent, with λ_max_ at 392–396 nm (Figure 2a). The log ε of **5a** was lower in ethanol than in other solvents (Table 1). By contrast, the absorption maxima of **5b**–**e**, which bear heterocycles, exhibited a significant bathochromic shift of greater than 60 nm compared with that of **5a**, as well as PNT (Figure 2b–f). These results were attributed to intramolecular charge transfer (ICT) to the extended π-conjugated system of naphtho[2,3-*d*]thiazole-4,9-dione skeleton arising from the electron-donating heterocyclic moiety. The observed bathochromic shifts in the absorption maxima of **5b**–**e** were affected by the polarity of the solvent (Table 1). In the polar solvent DMSO, a bathochromic shift of 13–15 nm was found when compared with the nonpolar solvent benzene. By contrast, as with PNT, no significant change was observed for the log ε of **5a**–**e** in either solvent.

**Figure 3:**
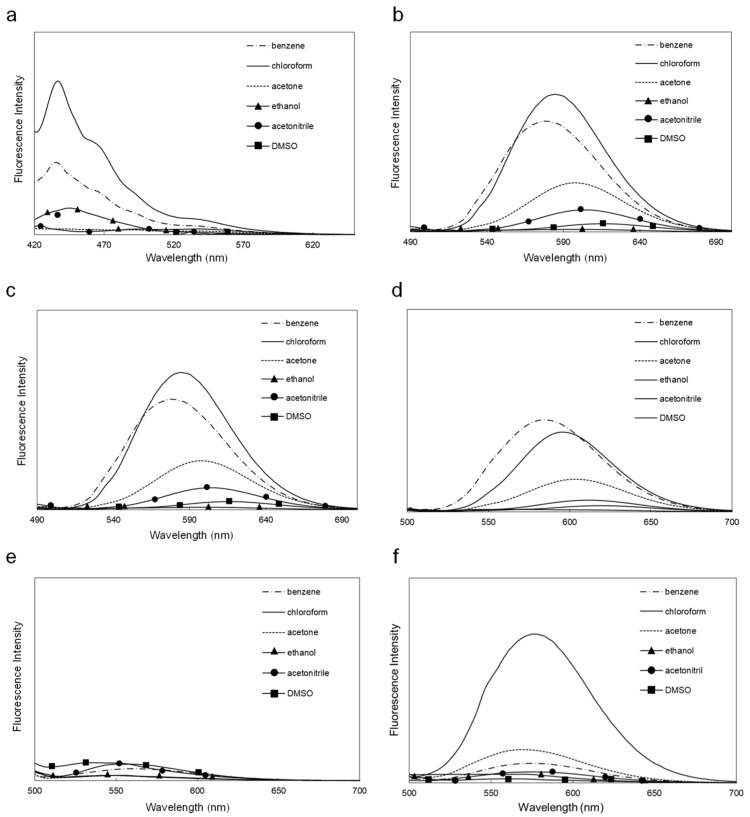
Fluorescence spectra of **5a**–**e** and **PNT** (**a**: **5a**, **b**: **5b**, **c**: **5c**, **d**: **5d**, **e**: **5e** and **f**: PNT) in benzene, chloroform, acetone, ethanol, acetonitrile, DMSO (1 × 10^−5^ mol/L).

**Figure 4:**
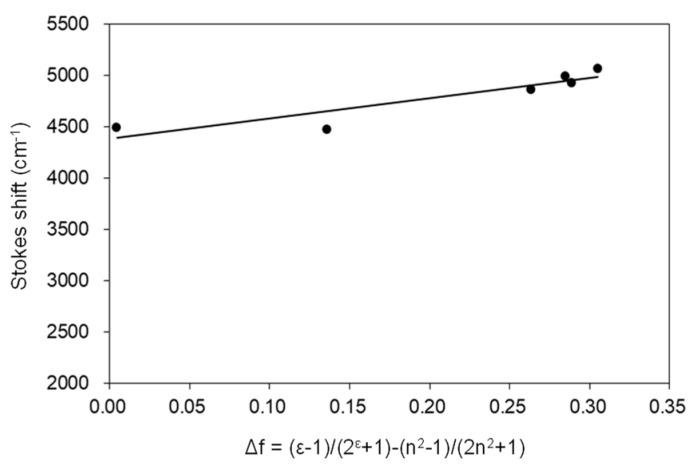
Lippert–Mataga plot of **5b** in various solvents. ε: dielectric constant, n: refractive index.

The fluorescence spectra of **5a**–**e** in solvents with different polarities are shown in Figure 3. Compound **5a** showed blue fluorescence at 436 nm in benzene, chloroform, acetone, and acetonitrile, but showed green fluorescence at 520 and 532 nm in ethanol and DMSO, respectively. In terms of Φ value, **5a** showed higher values in benzene and chloroform (0.10 and 0.17, respectively) than in the other solvents (Table 2). By contrast, the fluorescence spectra of **5b**–**e** and PNT, which bear heterocycles, exhibited a significant bathochromic shift of greater than 130 nm compared with that of **5a** in the nonpolar solvent benzene (Figure 3b–f). The Em_max_ of **5b**–**e** and PNT in benzene were in the range of 575–584 nm (Table 2). Interestingly, however, the Em_max_ of the PNT containing piperazine moiety did not change with solvent type, whereas a large bathochromic shift in fluorescence wavelengths was observed in **5b–e** with piperazine analogue (**5b**: morpholine, **5c**: thiomorpholine, **5d**: piperidine and **5e**: 4-methylpiperazine) as the solvent polarity increased (Table 2). The orange-red fluorescence of **5b**–**e** with Em_max_ of over 600 nm was observed in ethanol, acetonitrile, and DMSO. Piperazine, morpholine, thiomorpholine, piperidine, and 4-methylpiperazine are saturated nitrogen-containing heterocycles found in pharmaceuticals and bioactive substances. These heterocyclic rings are electron-donating groups and function as auxochromes, and are more water-soluble than aromatic rings because they exhibit a nonplanar, chair-shaped conformation. Compounds **5b**–**e** also exhibited a large Stokes shift greater than PNT, greater than 100 nm. The Stokes shifts of small fluorescence organic molecules are normally several tens of nm, and those exceeding 100 nm are often found in fluorescent lanthanide chelate complexes, fluorescent nanoparticles, and fluorescent proteins. Due to the fact that **5b**–**e** exhibits a large Stokes shift, specifically in polar solvents, it could be applied in biological imaging. The solvatochromic property of **5b**–**e** was also evaluated using a Lippert–Mataga plot, as shown in Figure 4 [25,26,27]. The Lippert–Mataga plot shows the relationship that exists between the solvent polarity parameter (Δf) and the Stokes shift of the absorption and emission maxima. The Stokes shifts revealed a linear relationship with the solvent polarity, thus suggesting that the dipole moment shows changes during excitation by ICT. The Φ values of **5b**–**d** depended on the polarity of the solvent. The Φ values of **5b**–**d** ranged from 0.04 to 0.13 in nonpolar solvents including benzene and chloroform, and the **5b** with morpholine group and **5c** with thiomorpholine group showed relatively large Φ values. Conversely, the Φ values of **5b**–**d** in acetone, ethanol, acetonitrile, and DMSO decreased significantly (Φ < 0.01) as the polarity of the solvent used in the measurement increased. The emission of ICT-based compounds was generally sensitive to the solvent polarity, and the Φ value decreased as the polarity of the solvent used in the measurement increased. The Φ values of **5e** and PNT, which bear the methylpiperazine or piperazine groups, were consistently low across all solvents investigated.

**Figure 5:**
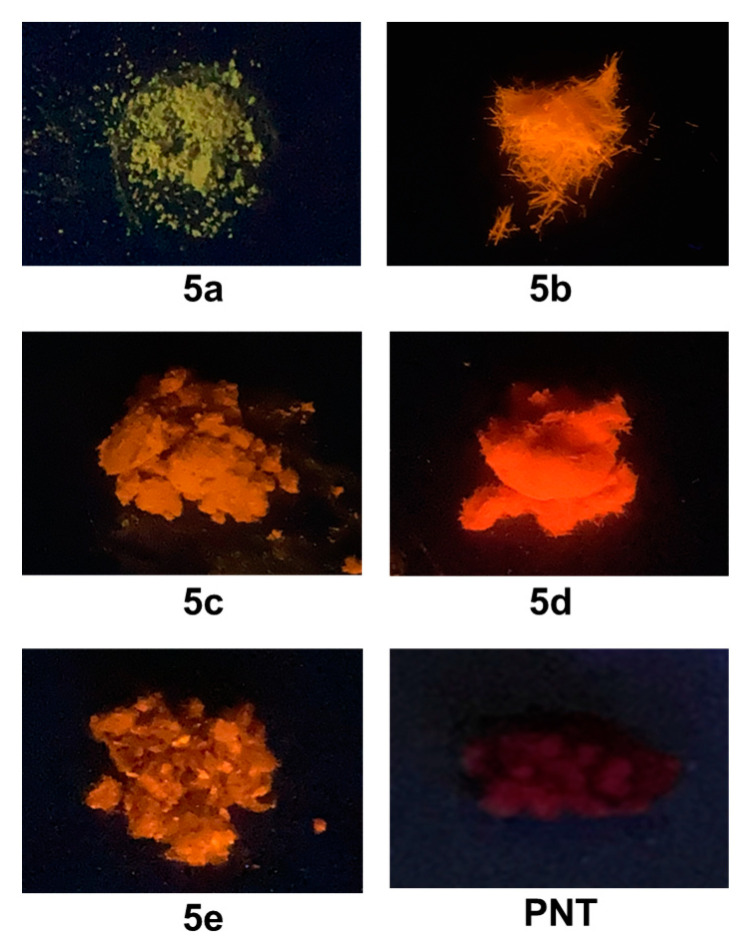
Solid state fluorescence photographs of **5a**–**e** and PNT irradiated with black light (365 nm).

Light emission in the solid state has attracted considerable attention due to its applicability in display materials, including optical sensors, organic light-emitting diodes, and information-recording machines [28,29]. In solution, each molecule is isolated by the solvent, whereas in the solid state, where molecules are aggregated, excimer formation and intermolecular energy transfer are more likely to occur, and the process followed by excited molecules is more favorable for quenching than for emission. Therefore, few molecules that show fluorescence both in solution and the solid state have been reported. However, it has recently been reported that, in small organic molecules, substituents can effectively affect the orientation and packing arrangement of the molecules by efficiently arranging them, resulting in the control of fluorescence properties such as emission wavelength and Φ values in the solid state [30,31,32]. Although no fluorescence was observed in the solid state for compounds **2** and **3**, which were used as raw materials in this study, **5a**–**e** and PNT with substituents at position 2 of the thiazole ring on naphtho[2,3-*d*]thiazole-4,9-dionen exhibited green-red fluorescence in the solid state (Figure 5). Table 3 lists Em_max_, Stokes shift, and Φ values of **5a**–**e** and PNT in solid state. The Em_max_ of **5a** was 564 m, whereas that of **5b**–**e** and PNT was on the longer wavelength side than **5a**. These results indicate that the direct bonding of the nitrogen-containing heterocyclic ring to naphtho[2,3-*d*]thiazole-4,9-dionen skeleton has a considerable effect on the orientation and packing of the entire molecule in the solid state. Among the **5b**–**e** and PNT, PNT with the piperazine group showed fluorescence at the longest wavelength side above 600 nm. Conversely, the Em_max_ in **5e** (577 nm) is approximately 50 nm shorter than that of PNT (628 nm), indicating that the methyl group at the nitrogen atom in the 4-position of the piperazine ring was not a major factor in the development of red fluorescent molecules. The Φ values of **5a**–**e** and PNT are low, ranging from 0.01 to 0.03, and it is difficult to improve the fluorescence intensity by the substituent at position 2, which was the subject of this study.

Compounds **2** and **3**, synthetic intermediates of **5a**–**e**, have been reported to be effective as antimicrobial agents for animals and wood preservatives [23,33,34]. In addition, the antimicrobial activity of **5a** against *Candida albicans*, *Aspergillus fumigatus*, *S. aureus*, and *S. epidermidis* has been reported [23]. Then, the antimicrobial activity of **5a**–**e** against *Staphylococcus* species (*S. aureus*, MRSA, and *S. epidermidis*) was evaluated using the micro-broth dilution method, in which antibiotics or antimicrobial substances are diluted and concentrated in steps and the MIC is measured. *Staphylococcus* are a large group of bacteria that can grow only in animals and humans [35]. *S. aureus* is a coagulase-positive and Gram-positive bacterium that is fungistatic under physiological conditions but transforms into serious pathogens under infection-promoting conditions [35]. MRSA is a methicillin-resistant *S. aureus*, whose virulence is comparable to that of *S. aureus*. However, MRSA is an important pathogen of nosocomial infections because it is resistant to various antimicrobial agents [36,37]. *S. epidermidis* is coagulase-negative and the most abundant bacterium on human skin, but it is usually non-pathogenic [35]. Table 4 lists the minimal inhibitory concentration values (µM) of **5a**–**e** against *Staphylococcus* (*S. aureus*, MRSA, and *S. epidermidis*). The antimicrobial activity of PNT is described in the previously reported data [22]. Compounds **5c** with thiomorpholine group and **5e** with 4-methylpiperazine group showed potent antimicrobial activity (15.8 and 8.0 µM, respectively) against *S. aureus*, although inferior to the antibacterial activity of PNT with piperazine group. Compounds **5c** and **5e** also demonstrated low MIC values (31.6 and 31.9 µM, respectively) for MRSA. Additionally, these compounds (**5c** and **5e**) showed potent inhibitory activity (15.8 and 8.0 µM, respectively) against *S. epidermidis*. By contrast, **5b** with morpholine group and **5d** with piperidine group showed no activity against *Staphylococcus*. These results suggest that the introduction of thiomorpholin, piperazine, and 4-methylpiperazine groups into naphtho[2,3-*d*]thiazole-4,9-diones confers antibacterial activity against various types of *Staphylococcus*, including MRSA, and the resultant compounds may be used as new antibacterial agents against *Staphylococcus* species. We intend to proceed with the synthesis of derivatives of **5c**, **5e,** and PNT to investigate their structure–activity relationships naphtho[2,3-*d*]thiazole-4,9-diones to bacteria in future research.

## 3. Materials and Methods

### 3.1. Reagents and Equipment

Chemical substances in this study were of analytic grade and were used without recrystallization or other purification. The identification and measurement of new compounds were performed using the following equipment. The melting point (MP) was determined by using Yanako MP-500D (Kyoto, Japan). The nuclear magnetic resonance (NMR) spectra were obtained using JEOL-JNM-EPC-400 (Tokyo, Japan) at 400 MHz and JEOL-JNM-ECA-500 (Tokyo, Japan) at 500 MHz. The JEOL JMS-700 mass spectrometer (Tokyo, Japan) was used to obtain the mass spectra (MS) and the high-resolution mass spectrometry (HRMS). Ultraviolet Visible (UV–Vis) absorption spectrum analysis was conducted using the Shimadzu UV-2450 spectrometer (Kyoto, Japan). Fluorescence spectrum analysis was conducted using the JASCO FP-8300 spectrometer (Tokyo, Japan). FTIR spectra were recorded on Shimadzu IRAffinity-1 (Kyoto, Japan). Three microbial strains were obtained from the Japan Collection of Microorganisms, Riken BioResource Research Center (Ibaraki, Japan), and the Biological Resource Center, National Institute of Technology and Evaluation (Tokyo, Japan). The strains used in this study were *S. aureus* (NRBC 12732), MRSA (JCM 16555), and *S. epidermidis* (NBRC 100911).

### 3.2. Synthesis of 2-(methylthio)naphtho[2,3-d]thiazole-4,9-dione (***2***)

First, 2-amino-3-chloronaphthalene-1,4-dione **1** (4.14 g, 16 mmol) and powdered sodium hydroxide (1.60 g, 40 mmol) were added to a 50 mL of DMSO at room temperature and stirred at 0 °C under a nitrogen atmosphere. Carbon disulfide (1.52 g, 20 mmol) was slowly added to the above solution for 20 min, and the solution was stirred for 1 h under 10 °C. After stirring at room temperature for another 3 h, 3.15 g of dimethyl sulfate (25 mmol) was added slowly for 30 min and stirred for another 1 h. After adding 200 mL of cold water and neutralizing using 10% HCl solution, the resulting yellow precipitate was obtained and washed several times with cold water. Recrystallization using MeOH produced the desired compound **2** (4.74 g, 18 mmol) as orange needles in a 91% yield. MP: 208–209 °C. ^1^H NMR (DMSO-d_6_, 400 MHz) δ: 2.84 (3H, s), 7.87–7.93 (2H, m), 8.08–8.16 (2H, m). MS *m*/*z*: 260 [M^+^]. HRMS calcd for C_12_H_7_NO_2_S_2_ [M^+^]: 260.9918. Found: 260.9919.

### 3.3. Synthesis of 2-(methylsulfinyl)naphtho[2,3-d]thiazole-4,9-dione (***3***)

First, 1.72 g of m-chloroperoxybenzoic acid (10 mmol) was added to a 20 mL solution of dichloromethane dissolved with 2.60 g of 2-(methylthio)naphtho[2,3-*d*]thiazole-4,9-dione (**2**) (10 mmol) and stirred at room temperature for 2 h. Residues were collected by filtration method. After drying, the formed product was obtained through recrystallization with MeOH to obtain the desired compound **3** (1.87 g, 6.8 mmol) as yellow crystals in a 68% yield. Mp: 247–248 °C. ^1^H NMR (DMSO-d_6_, 400 MHz) δ: 3.65 (3H, s), 7.95–8.01 (2H, m), 8.16–8.25 (2H, m). ^13^C NMR (DMSO-d_6_, 125 MHz) δ: 42.1, 126.7, 127.3, 132.6, 134.5, 135.1, 145.7, 153.9, 171.4, 176.6, 177.8, 186.5. MS *m*/*z*: 277 [M^+^]. HRMS calcd for C_12_H_7_NO_3_S_2_ [M^+^]: 276.9867. Found: 276.9869.

### 3.4. Synthesis of N-(4,9-dioxo-4,9-dihydronaphtho[2,3-d]thiazol-2-yl)benzamide (***5a***)

The mixed solution of 2-(methylsulfinyl)naphtho[2,3-*d*]thiazole-4,9-dione (**3**) (0.26 g, 1.0 mmol) with 0.21 g of benzylamine **4a** (2.0 mmol) was reacted at 100 °C for 2 h. After cooling the reacting solution, 10 mL of MeOH was added at room temperature. The residue was obtained through filtration. Recrystallization using MeOH produced the desired compound **5a** (0.21 g, 0.6 mmol) as orange needles in a 64% yield. Mp: 280–281 °C. ^1^H NMR (DMSO-d_6_, 400 MHz) δ: 7.60 (2H, t, *J* = 7.6 Hz), 7.70 (1H, t, *J* = 7.6 Hz), 7.90 (2H, t, *J* = 4.6 Hz), 8.10–8.20 (4H, m). IR (potassium bromide) ν_max_ cm^−1^: 3160, 3070, 1666, 1640. MS *m*/*z*: 334 [M^+^]. HRMS calcd for C_18_H_10_N_2_O_3_S [M^+^]: 334.0412. Found: 334.0410.

### 3.5. Synthesis of 2-morpholinonaphtho[2,3-d]thiazole-4,9-dione (***5b***)

**5b** (0.21 g, 0.7 mmol) was synthesized in 70% yield from 2-(methylsulfinyl)naphtho[2,3-*d*]thiazole-4,9-dione (**3**) (0.26 g, 1.0 mmol) with 0.17 g of morpholine **4b** (2.0 mmol) using a similar method to that used for **5a**. Recrystallization with MeOH produced orange needles. Mp: 307–308 °C. ^1^H NMR (DMSO-d_6_, 400 MHz) δ: 3.67 (4H, t, *J* = 4.0, 5.2 Hz), 3.76 (4H, t, *J* = 4.4, 5.2 Hz), 7.83–7.85 (2H, m), 8.01–8.08 (2H, m). ^13^C NMR (DMSO-d_6_, 125 MHz) δ: 48.4, 65.2, 125.6, 126.7, 130.9, 131.9, 132.8, 133.7, 134.0, 154.0, 173.4, 176.8, 177.6. IR (potassium bromide) ν_max_ cm^−1^: 1675, 1640. MS *m*/*z*: 300 [M^+^]. HRMS calcd for C_15_H_12_N_2_O_3_S [M^+^]: 300.0569. Found: 300.0566.

### 3.6. Synthesis of 2-thiomorpholinonaphtho[2,3-d]thiazole-4,9-dione (***5c***)

**5c** (0.19 g, 0.6 mmol) was synthesized in 60% yield from 2-(methylsulfinyl)naphtho[2,3-*d*]thiazole-4,9-dione (**3**) (0.26 g, 1.0 mmol) with 0.21 g of thiomorpholine **4c** (2.0 mmol) using a similar method to that used for **5a**. Recrystallization with MeOH produced orange needles. Mp: 245–246 °C. ^1^H NMR (DMSO-d_6_, 400 MHz) δ: 2.81 (2H, t, *J* = 3.9, 5.2 Hz), 3.97 (2H, t, *J* = 4.8, 5.2 Hz), 7.83–7.93 (2H, m), 8.00–8.16 (2H, m). ^13^C NMR (DMSO-d_6_, 125 MHz) δ: 26.0, 51.4, 126.3, 127.0, 130.8, 132.2, 132.5, 134.2, 134.5, 154.0, 176.6, 176.8, 177.3. IR (potassium bromide) ν_max_ cm^−1^: 1670, 1640. MS *m*/*z*: 316 [M^+^]. HRMS calcd for C_15_H_12_N_2_O_2_S_2_ [M ^+^]: 316.0340. Found: 316.0340.

### 3.7. Synthesis of 2-(piperidin-1-yl)naphtho[2,3-d]thiazole-4,9-dione (***5d***)

**5d** (0.21 g, 0.7 mmol) was synthesized in 70% yield from 2-(methylsulfinyl)naphtho[2,3-*d*]thiazole-4,9-dione (**3**) (0.26 g, 1.0 mmol) with 0.17 g of piperidine **4d** (0.17 g, 2.0 mmol) using a similar method to that used for **5a**. Recrystallization with MeOH produced orange needles. Mp: 221–222 °C. ^1^H NMR (DMSO-d_6_, 400 MHz) δ: 1.65 (6H, m), 3.67 (4H, m), 7.80–7.85 (2H, m), 7.99–8.06 (2H, m). ^13^C NMR (DMSO-d_6_, 125 MHz) δ: 23.1, 24.7, 49.6, 125.4, 126.6, 130.2, 131.9, 132.8, 133.5, 133.9, 154.3, 172.8, 176.5, 177.6. IR (potassium bromide) ν_max_ cm^−1^: 1675, 1636. MS *m*/*z*: 298 [M+]. HRMS calcd for C_16_H_14_N_2_O_2_S [M^+^]: 298.0776. Found: 298.0776.

### 3.8. Synthesis of 2-(4-methylpiperazin-1-yl)naphtho[2,3-d]thiazole-4,9-dione (***5e***)

**5e** (0.21 g, 0.7 mmol) was synthesized in 68% yield from 2-(methylsulfinyl)naphtho[2,3-*d*]thiazole-4,9-dione (**3**) (0.26 g, 1.0 mmol) with 0.20 g of piperidine **4e** (2.0 mmol) using a similar method to **5a**. Recrystallization with MeOH produced orange needles. Mp: 226–227 °C. ^1^H NMR (400 MHz, DMSO-d_6_) δ: 2.25 (3H, s), 2.47 (4H, t, *J* = 4.9 Hz), 3.67 (4H, t, *J* = 4.8 Hz), 7.82–7.92 (2H, m), 8.00–8.07 (2H, m). ^13^C NMR (DMSO-d_6_, 125 MHz) δ: 36.0, 48.4, 53.5, 125.6, 126.7, 130.8, 131.9, 132.8, 133.7, 134.0, 154.2, 173.0, 176.8, 177.7. IR (potassium bromide) ν_max_ cm^−1^: 1675, 1642. MS *m*/*z*: 313 [M^+^]. HRMS calcd for C_16_H_15_N_3_O_2_S [M^+^]: 313.0885. Found: 313.0884.

### 3.9. Synthesis of 2-(piperazin-1-yl)naphtho[2,3-d]thiazole-4,9-dione (PNT)

PNT was prepared according to previous reports [22]. PNT (0.26 g, 1.0 mmol) was obtained from 0.26 g of 2-(methylsulfinyl)naphtho[2,3-*d*]thiazole-4,9-dione (1.0 mmol) with 0.17 g of piperazine (2.0 mmol) in 50% yield. Mp: 214–215 °C. ^1^H NMR (DMSO-d_6_, 400 MHz) δ: 2.87 (4H, t, *J* = 5.2 Hz), 3.62 (4H, t, *J* = 4.8 Hz), 7.82–7.86 (2H, m), 7.99–8.07 (2H, m). ^13^C NMR δ: (DMSO-d_6_, 125 MHz) δ 44.5, 49.5, 125.5, 126.6, 130.4, 131.9, 132.7, 133.5, 133.9, 154.1, 173.1, 176.6, 177.6. IR (potassium bromide) ν_max_ cm^−1^: 1670, 1637. MS *m*/*z*, 299 [M^+^]. HRMS calculated for C_15_H_13_N_3_O_2_S [M^+^], 299.0728; found, 299.0727.

### 3.10. UV–Vis Absorption Measurements

The compounds (**5a**–**e** and PNT) were dissolved using dimethyl sulfoxide to obtain 10^−2^ mol/L of stock solutions. Thereafter, 10^−4^ mol/L solutions of **5a**–**e** and PNT were prepared using various solvents (benzene, chloroform, acetone, ethanol, acetonitrile, and DMSO) prior to UV–Vis absorption measurement. After the solution was placed in a cuvette, UV–Vis absorption spectrum was measured through scanning from 300 nm to 600 nm.

### 3.11. Fluorescence Measurements

In solution: The compounds (**5a**–**e** and PNT) were dissolved using DMSO to obtain 10^−2^ mol/L of stock solutions. Thereafter, 10^−5^ mol/L solution of **5a**–**e** and PNT were prepared using various solvents (benzene, chloroform, acetone, ethanol, acetonitrile, and DMSO) prior to fluorescence measurement. After the solution was placed in a quartz cell and set in a fluorescence spectrometer, the excitation wavelength of the evaluated compounds was measured by investigating their emission wavelength. Similarly, the emission wavelength of the evaluated compounds was determined by determining their excitation wavelength. The exact excitation and emission wavelengths of each compound were obtained by repeating this operation. The fluorescence quantum yield (Φ) in the solution was measured using Rhodamine B as a standard (Φ = 0.7 in ethanol). In the solid state: Approximately 10 mg of the compound was placed on a cell for solid-state fluorescence measurement, and excitation and emission wavelengths of each compound were determined in the same procedure as for measurements in the solution. The fluorescence quantum yield (Φ) in solid state was conducted using Hamamastu Photonics Absolute PL Quantum Yield Measurement System C9920-01 (Shizuoka, Japan).

### 3.12. Antimicrobial Assays

The minimum inhibitory concentration (MIC) was used to determine the antibacterial activity of each compound. Compounds were dissolved in DMSO, and the final concentrations of each compound were 0, 0.2, 0.3, 0.6, 1.3, 2.5, 5, 10, 20, 40, and 80 µg/mL. *Staphylococcus* strains (*S. aureus*, MRSA, and *S. epidermidis*) in the exponential growth condition were diluted in a Mueller–Hinton broth to a concentration of 4 × 10^4^ CFU/mL. Thereafter, the culture medium (150 µL) was dispensed into a 96-well microtiter plate. The susceptibility test was conducted by using two-fold standard broth microdilutions of **5a**–**e** according to Clinical and Laboratory Standards Institute guidelines. Samples with concentrations ranging from 0.12 to 268.5 µM were used to determine the MIC. Each strain was cultivated for two days at 37 °C. Each experiment was conducted thrice. The MIC was defined as the lowest concentration (highest dilution concentration) required to inhibit microbial growth.

## 4. Conclusions

We successfully synthesized novel naphtho[2,3-*d*]thiazole-4,9-dione compounds (**5a**–**e**) in moderate-to-good yields via the reaction of **3** with amines, including benzylamine (**4a**), morpholine (**4b**), thiomorpholine (**4c**), piperidine (**4d**), and 4-methylpiperazine (**4e**), and evaluated their absorption and fluorescence properties in solution and the solid state and antimicrobial activities against *Staphylococcus* species (*S. aureus*, MRSA, and *S. epidermidis*). In solution, all compounds showed fluorescence; specifically, **5b**–**e** bearing a nitrogen-containing heterocyclic ring at the 2-position of the thiazole ring exhibited a significant bathochromic shift of greater than 130 nm that of **5a** with benzylamine group in the nonpolar solvent benzene, as well as the PNT with piperazine group. A solvatochromic effect was also observed, and **5b**–**e** exhibited orange-red fluorescence above 600 nm in highly polar ethanol, acetonitrile, and DMSO. In the solid state, as in solution, **5b**–**e** and PNT showed fluoresce at longer wavelengths than **5a**, suggesting that direct nitrogen-containing heterocyclic bonding to the naphtho[2,3-*d*]thiazole-4,9-dionen skeleton has a significant effect on the fluorescence of the naphtho[2,3-*d*]thiazole-4,9-dionen compound. Among the compounds prepared, **5c** with thiomorpholine group and **5e** with 4-methylpiperazine group showed potent inhibitory activity against *Staphylococcus* species (*S. aureus*, MRSA, and *S. epidermidis*), although inferior to the antibacterial activity of PNT. These findings suggest that the introduction of nitrogen-containing heterocycles such as thiomorpholin, piperazine, and 4-methylpiperazine groups into naphtho[2,3-*d*]thiazole-4,9-diones is effective for the development of antibacterial activity based on naphtho[2,3-*d*]thiazole-4,9-dione, and provide valuable information for the creation of fluorescence materials with antimicrobial activity.

## Figures and Tables

**Table 1:** UV–Vis absorption data of **5a**–**e** and PNT in various solvents.

Absorption Maximum (nm) (log ε)						
Solvent	5a	5b	5c	5d	5e	PNT
benzene	396 (3.36)	460 (3.63)	461 (3.48)	471 (3.63)	464 (3.56)	468 (3.27)
chloroform	392 (3.32)	463 (3.62)	464 (3.49)	480 (3.62)	468 (3.55)	464 (3.25)
acetone	392 (3.38)	462 (3.62)	464 (3.50)	473 (3.63)	466 (3.58)	470 (3.27)
ethanol	392 (2.24)	463 (3.59)	464 (3.35)	476 (3.60)	465 (3.54)	473 (3.27)
acetonitrile	396 (3.38)	464 (3.62)	467 (3.49)	477 (3.64)	468 (3.58)	470 (3.26)
DMSO	396 (3.40)	474 (3.63)	476 (3.53)	484 (3.63)	477 (3.57)	483 (3.25)

**Table 2:** Fluorescence data of **5a**–**e** and **PNT** in various types of solvents.

Emission Maximum (nm) (Φ ^a^), SS ^b^ (nm)						
Solvent	5a	5b	5c	5d	5e	PNT
benzene	436 (0.10),	580 (0.07),	575 (0.12),	584 (0.05),	584 (<0.01),	575 (<0.01),
	40	120	114	113	116	107
chloroform	436 (0.17),	584 (0.08),	582 (0.13),	597 (0.04),	597 (<0.01),	576 (0.03),
	44	121	118	117	133	112
acetone	436 (0.01),	599 (0.02),	596 (0.03),	605 (0.01),	605 (<0.01),	576 (<0.01),
	44	137	132	132	135	106
ethanol	520 (0.01),	600 (0.01),	602 (0.02),	604 (0.01),	604 (<0.01),	573 (<0.01),
	128	137	138	128	131	100
acetonitrile	436 (<0.01),	605 (0.01),	602 (0.01),	614 (<0.01),	614 (<0.01),	577 (<0.01),
	40	141	135	137	144	107
DMSO	532 (0.03),	616 (<0.01),	611 (0.01),	622 (<0.01),	622 (<0.01),	577 (<0.01),
	176	142	135	138	139	94

^a^ Quantum yields were measured using rhodamine B as standard (Φ = 0.7 in ethanol). ^b^ Stokes shift, Em_max_—Ex_max_.

**Table 3:** Solid state fluorescence data of **5a**–**e** and PNT.

Compounds	Ex_max_ (nm)	Em_max_ (nm)	SS ^a^	Φ ^b^
**5a**	427	564	137	0.01
**5b**	375	582	207	0.03
**5c**	400	579	179	0.01
**5d**	518	596	78	0.01
**5e**	521	577	56	0.01
PNT	526	628	102	0.01

^a^ Stokes shift, Em_max_—Ex_max_. ^b^ Quantum yields were measured by using Absolute PL Quantum Yield Measurement System (C9920-01).

**Table 4:** Minimum inhibitory concentration (MIC) of **5a**–**e** and PNT against *S. aureus*, MRSA, and *S. epidermidis*.

Compounds		MIC (μg/mL)	
	*S. aureus*	MRSA	*S. epidermidis*
**5a**	119.7 ± 0.0	119.7 ± 0.0	119.7 ± 0.0
**5b**	133.3 ± 0.0	133.0 ± 0.0	133.3 ± 0.0
**5c**	15.8 ± 0.0	31.6 ± 0.0	15.8 ± 0.0
**5d**	134.2 ± 0.0	134.2 ± 0.0	134.2 ± 0.0
**5e**	8.0 ± 0.0	31.9 ± 0.0	16.0 ± 0.0
PNT [22]	2.5 ± 0.0	6.7 ± 2.9	2.5 ± 0.0

Results are represented as mean ± S.D.

## Data Availability

The data presented in this study are available in this article.

## Supplementary Materials

The following supporting information can be downloaded at: https://www.mdpi.com/article/10.3390/molecules29122777/s1, Figure S1: HPLC chromatogram of **5a**; Figure S2: HPLC chromatogram of **5b**; Figure S3: HPLC chromatogram of **5c**; Figure S4: HPLC chromatogram of **5d**; Figure S5: HPLC chromatogram of **5e**; Figure S6: HPLC chromatogram of PNT; Figure S7: ^1^H NMR spectrum (400 MHz, DMSO-d_6_) of **2**; Figure S8: ^1^H NMR spectrum (400 MHz, DMSO-d_6_) of **3**; Figure S9: ^13^C NMR spectrum (125 MHz, DMSO-d_6_) of **3**; Figure S10: ^1^H NMR spectrum (400 MHz, DMSO-d_6_) of **5a**; Figure S11: ^1^H NMR spectrum (400 MHz, DMSO-d_6_) of **5b**; Figure S12: ^13^C NMR spectrum (125 MHz, DMSO-d_6_) of **5b**; Figure S13: ^1^H NMR spectrum (400 MHz, DMSO-d_6_) of **5c**; Figure S14: ^13^C NMR spectrum (125 MHz, DMSO-d_6_) of **5c**; Figure S15: ^1^H NMR spectrum (400 MHz, DMSO-d_6_) of **5d**; Figure S16: ^13^C NMR spectrum (125 MHz, DMSO-d_6_) of **5d**; Figure S17: ^1^H NMR spectrum (400 MHz, DMSO-d_6_) of **5e**; Figure S18: ^13^C NMR spectrum (125 MHz, DMSO-d_6_) of **5e**; Figure S19: ^1^H NMR spectrum (400 MHz, DMSO-d_6_) of PNT; Figure S20: ^13^C NMR spectrum (125 MHz, DMSO-d_6_) of PNT.
