# Supplementary material for: Synthesis and Photophysical Characterization of Fluorescent Naphtho[2,3-d]thiazole-4,9-Diones and Their Antimicrobial Activity against Staphylococcus Strains
Source: Molecules. 2024 Jun 11;29(12):2777. doi: 10.3390/molecules29122777 (PMC11206905; doi:10.3390/molecules29122777)
Supplement: Supplementary file 1 [file molecules-29-02777-s001.zip › molecules-3004104-supplementary.pdf]

Supplementary Materials

Synthesis and photophysical characterization of fluorescent naphtho[2,3-d]thiazole-4,9-diones and their antimicrobial activity against *Staphylococcus* strains

Masayori Hagimori [1,*], Fumiko Hara [1], Naoko Mizuyama [2], Shinya Takada [1], Saki Hayashi [3], Tamami Haraguchi [3,4], Yoshiro Hatanaka [5], Toshihiro Nagao [5], Shigemitsu Tanaka [5], Miki Yoshii [5] and Miyako Yoshida [3,4,*]

[1]Department of Analitical Chemistry, Faculty of Pharmaceutical Sciences, Mukogawa Women's University, 11-68 Koshien 9-Bancho, Nishinomiya City 663-8179, Hyogo, Japan

[2]Division of Medical Innovation, Translational Research Center for Medical Innovation, 1-5-4 Minatoji-ma-minamimachi, Chuo-ku, Kobe 650-0047, Japan; hagimori@fbri.org

[3]Department of Clinical Pharmaceutics, Faculty of Pharmaceutical Sciences, Mukogawa Women's University, 11-68 Koshien 9-Bancho, Nishinomiya City 663-8179, Hyogo, Japan

[4]Institute for Women's Career Advancement and Gender Equality Development, Mukogawa Women's University, 6-46 Ikebiraki, Nishinomiya, Hyogo 663-8558, Japan

[5]Osaka Research Institute of Industrial Science and Technology, 1-6-50 Morinomiya, Joto-ku, Osaka City 536-8553, Osaka, Japan

Contents

1. Figure S1. HPLC chromatogram of **5a**

2. Figure S2. HPLC chromatogram of **5b**

3. Figure S3. HPLC chromatogram of **5c**

4. Figure S4. HPLC chromatogram of **5d**

5. Figure S5. HPLC chromatogram of **5e**

6. Figure S6. HPLC chromatogram of **PNT**

7. Figure S7. ^{1}H NMR spectrum (400 MHz, DMSO-d_6) of **2**

8. Figure S8. ^{1}H NMR spectrum (400 MHz, DMSO-d_6) of **3**

9. Figure S9. ^{13}C NMR spectrum (125 MHz, DMSO-d_6) of **3**

10. Figure S10. ^{1}H NMR spectrum (400 MHz, DMSO-d_6) of **5a**

11. Figure S11. ^{1}H NMR spectrum (400 MHz, DMSO-d_6) of **5b**

12. Figure S12. ^{13}C NMR spectrum (125 MHz, DMSO-d_6) of **5b**

13. Figure S13. ^{1}H NMR spectrum (400 MHz, DMSO-d_6) of **5c**

14. Figure S14. ^{13}C NMR spectrum of (125 MHz, DMSO-d_6) **5c**

15. Figure S15. ^{1}H NMR spectrum (400 MHz, DMSO-d_6) of **5d**

16. Figure S16. ^{13}C NMR spectrum (125 MHz, DMSO-d_6) of **5d**

17. Figure S17. ^{1}H NMR spectrum (400 MHz, DMSO-d_6) of **5e**

18. Figure S18. ^{13}C NMR spectrum (125 MHz, DMSO-d_6) of **5e**

19. Figure S19. ^{1}H NMR spectrum (400 MHz, DMSO-d_6) of **PNT**

20. Figure S20. ^{13}C NMR spectrum (125 MHz, DMSO-d_6) of **PNT**

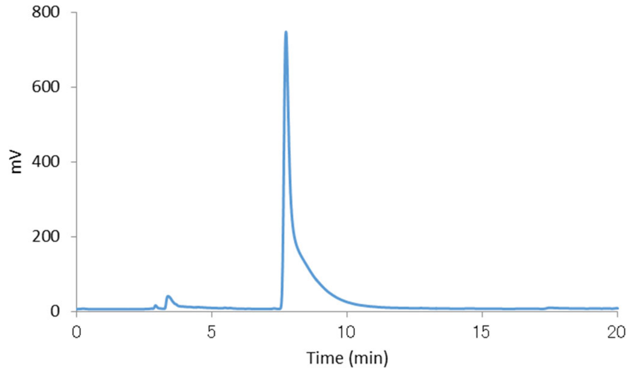

Figure S1. HPLC chromatogram of **5a**. HPLC condition; column: COSMOSIL 5C18-AR-II (4.6 x 250 mm), flow rate: 1 mL/min, temp: 25 °C, eluent: 0.05 % TFA aq. : CH_3CN = 20 : 80, UV: 254 nm, retention time: 7.73 min.

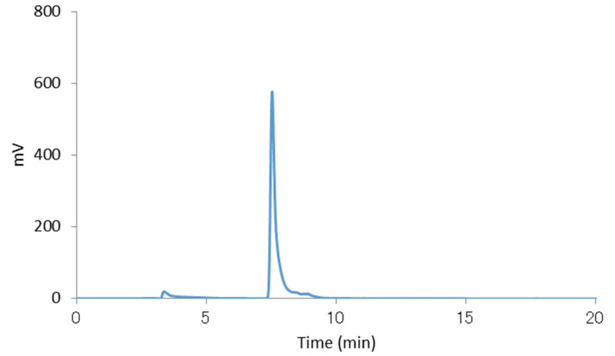

Figure S2. HPLC chromatogram of **5b**. HPLC condition; column: COSMOSIL 5C18-AR-II (4.6 x 250 mm), flow rate: 1 mL/min, temp: 25 °C, eluent: 0.05 % TFA aq. : CH_3CN = 30 : 70, UV: 254 nm, retention time: 7.52 min.

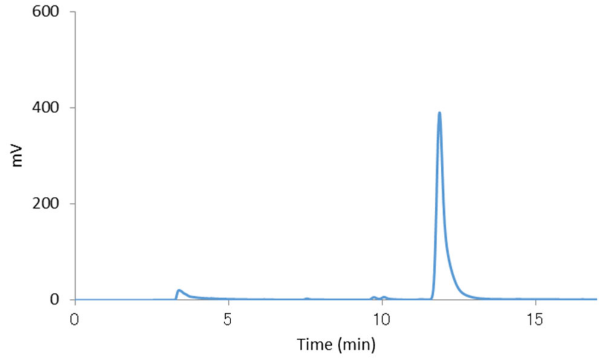

Figure S3. HPLC chromatogram of **5c**. HPLC condition; column: COSMOSIL 5C18-AR-II (4.6 x 250 mm), flow rate: 1 mL/min, temp: 25 °C, eluent: 0.05 % TFA aq. : CH_3CN = 30 : 70, UV: 254 nm, retention time: 11.87 min.

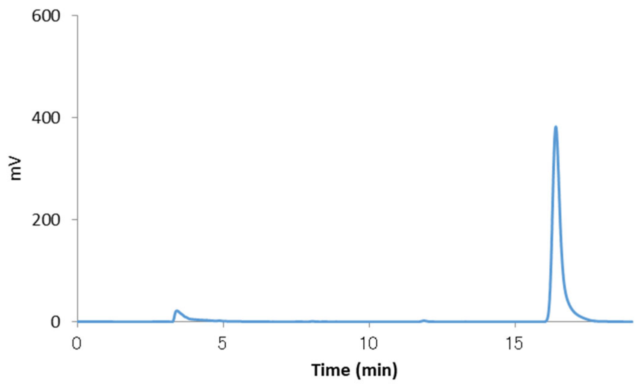

Figure S4. HPLC chromatogram of **5d**. HPLC condition; column: COSMOSIL 5C18-AR-II (4.6 x 250 mm), flow rate: 1 mL/min, temp: 25 °C, eluent: 0.05 % TFA aq. : CH_3CN = 30 : 70, UV: 254 nm, retention time: 16.38 min.

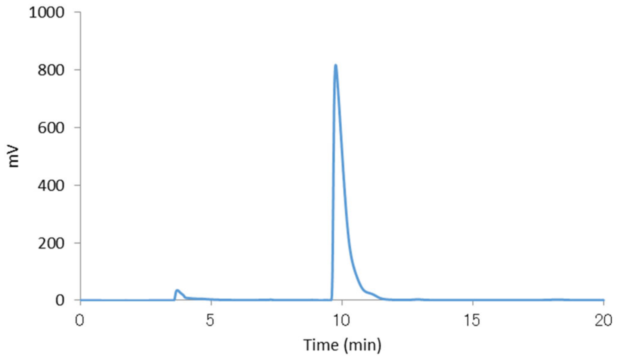

Figure S5. HPLC chromatogram of **5e**. HPLC condition; column: COSMOSIL 5C18-AR-II (4.6 x 250 mm), flow rate: 1 mL/min, temp: 25 °C, eluent: 0.05 % TFA aq. : CH_3CN = 55 : 45, UV: 254 nm, retention time: 9.76 min.

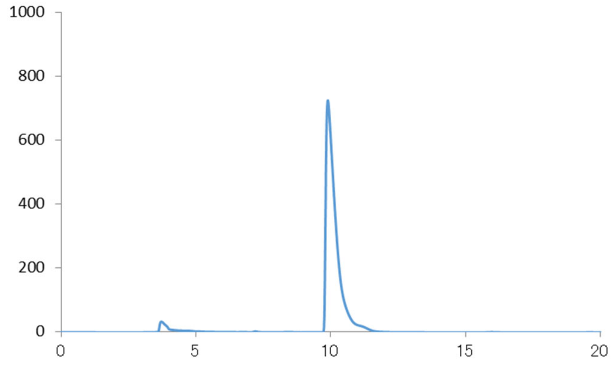

Figure S6. HPLC chromatogram of **PNT**. HPLC condition; column: COSMOSIL 5C18-AR-II (4.6 x 250 mm), flow rate: 1 mL/min, temp: 25 °C, eluent: 0.05 % TFA aq. : CH_3CN = 55 : 45, UV: 254 nm, retention time: 9.89 min.

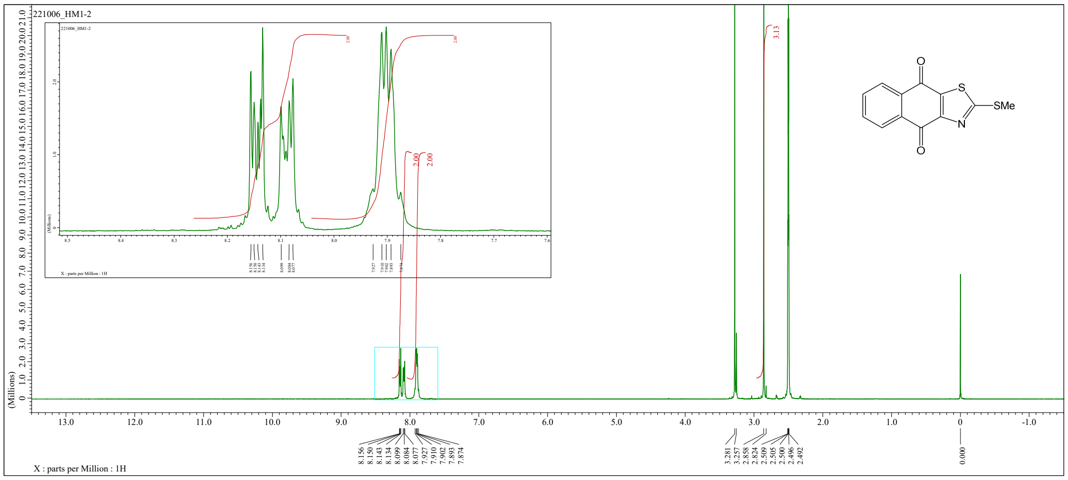

Figure S7. ^{1}H NMR spectrum (400 MHz, DMSO-d_6) of **2**

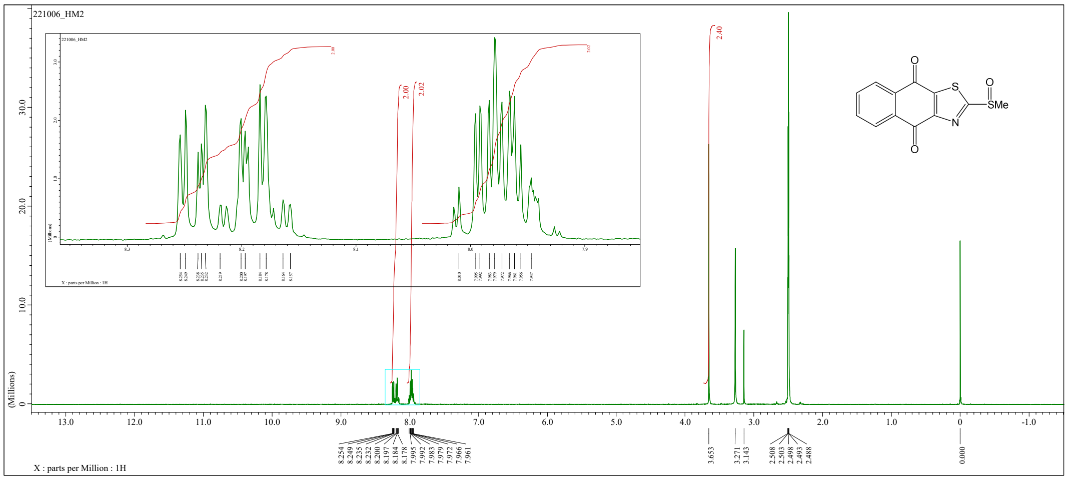

Figure S8. ^{1}H NMR spectrum (400 MHz, DMSO-d_6) of **3**

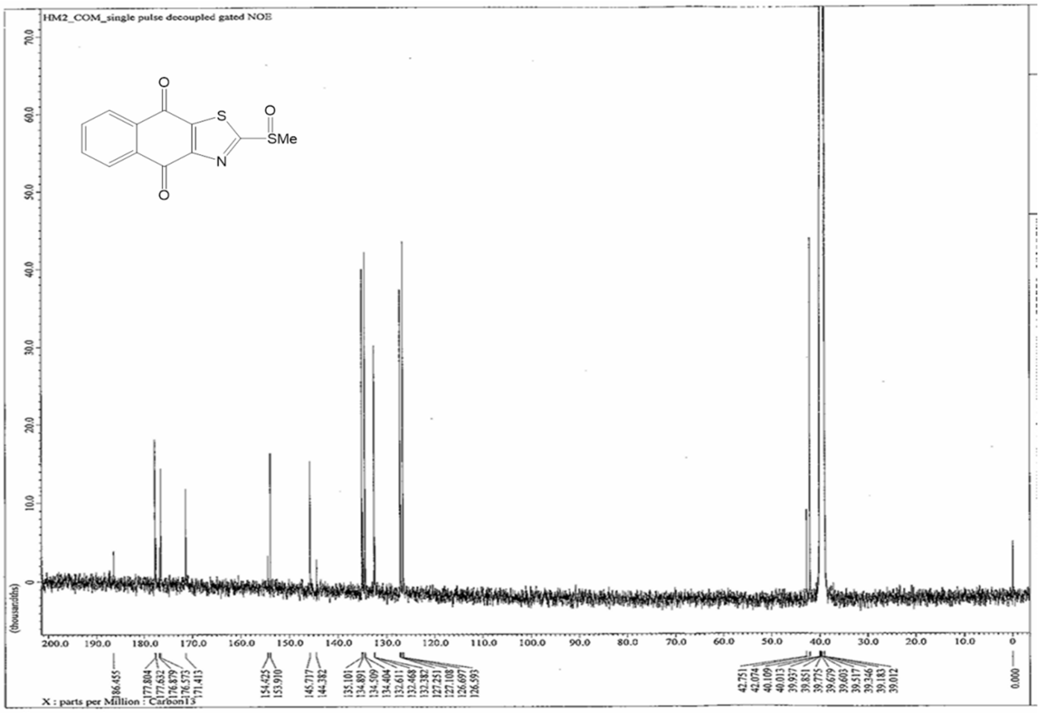

Figure S9. ^{13}C NMR spectrum (125 MHz, DMSO-d_6) of **3**

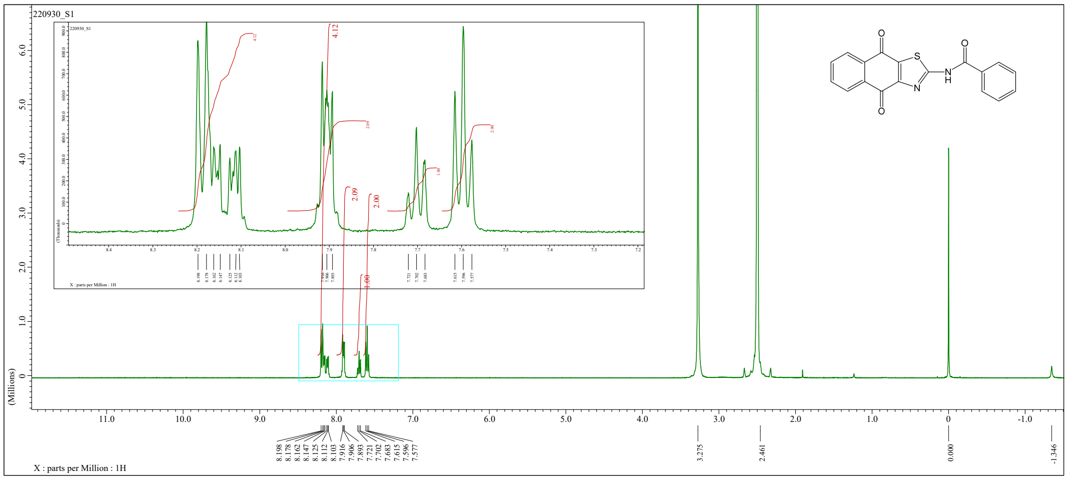

Figure S10. ^{1}H NMR spectrum (400 MHz, DMSO-d_6) of **5a**

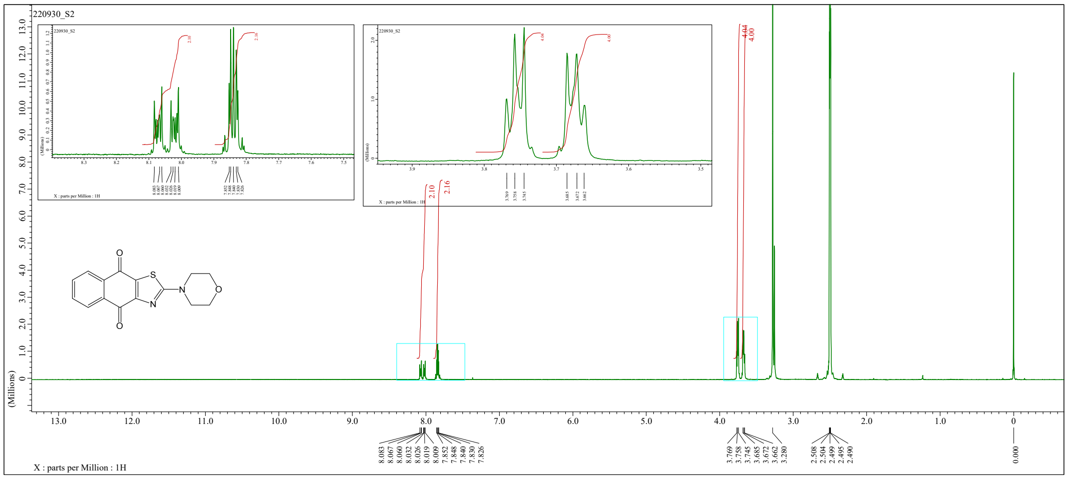

Figure S11. ^{1}H NMR spectrum (400 MHz, DMSO-d_6) of **5b**

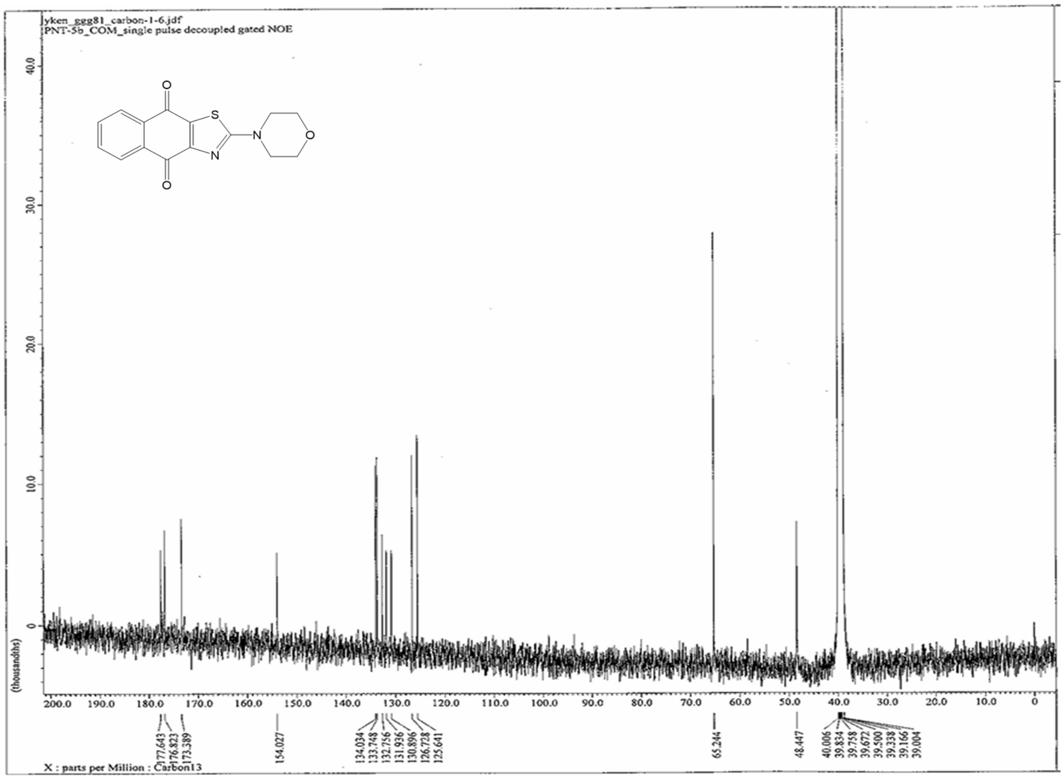

Figure S12. ^{13}C NMR spectrum (125 MHz, DMSO-d_6) of **5b**

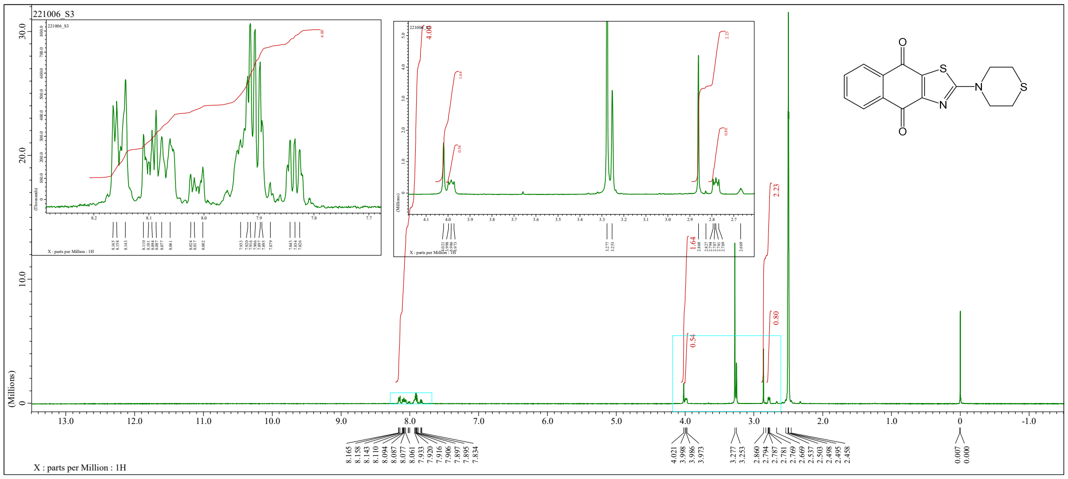

Figure S13. ^{1}H NMR spectrum (400 MHz, DMSO-d_6) of **5c**

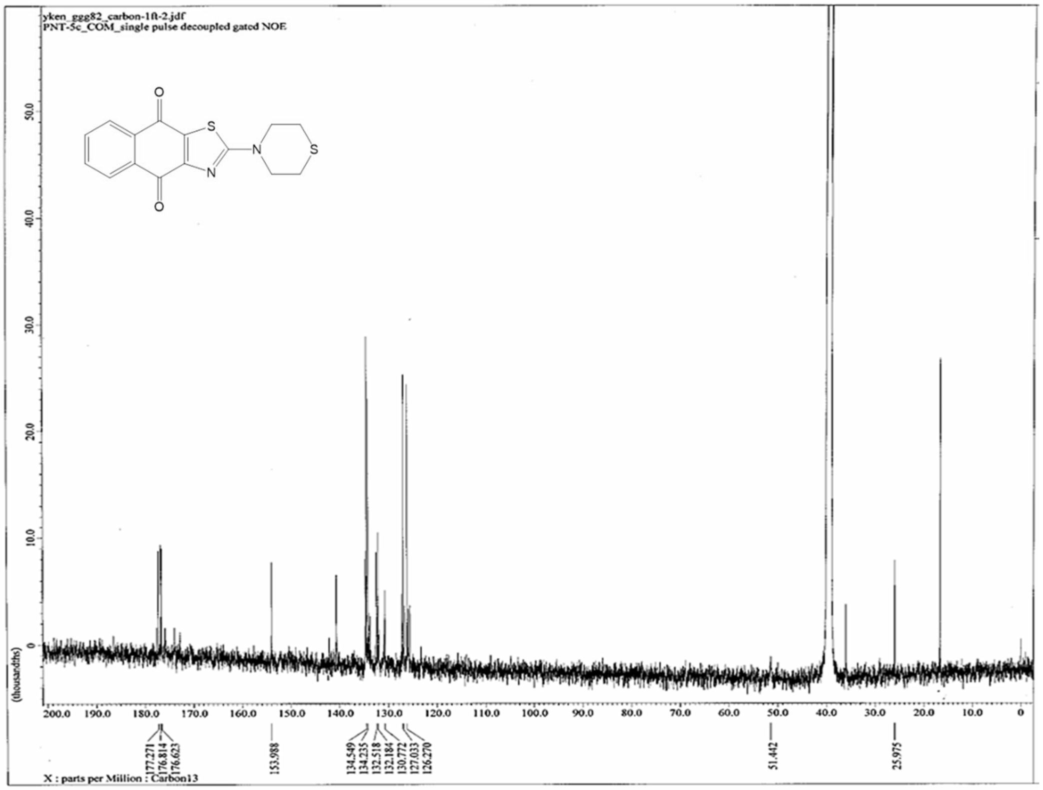

Figure S14. ^{13}C NMR spectrum (125 MHz, DMSO-d_6) of **5c**

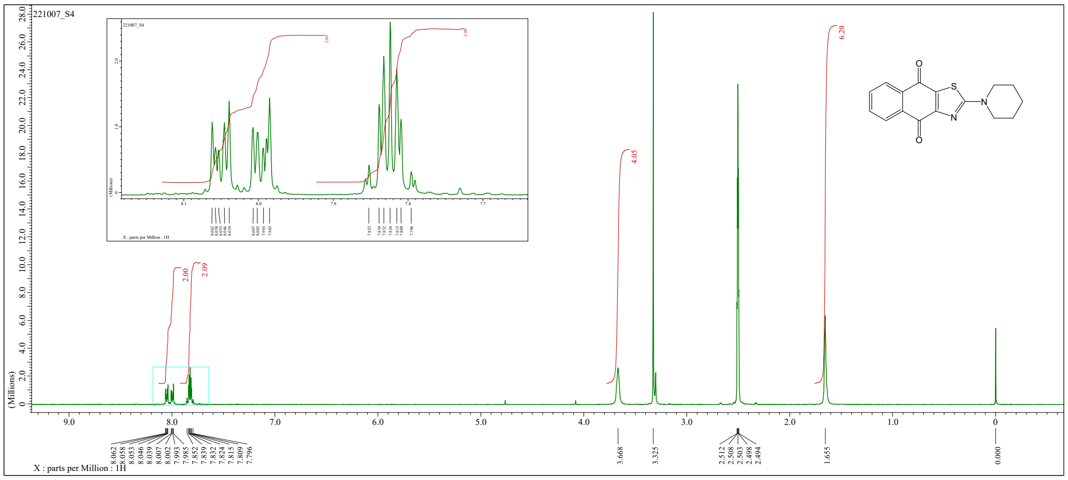

Figure S15. ^{1}H NMR spectrum (400 MHz, DMSO-d$_6$) of **5d**

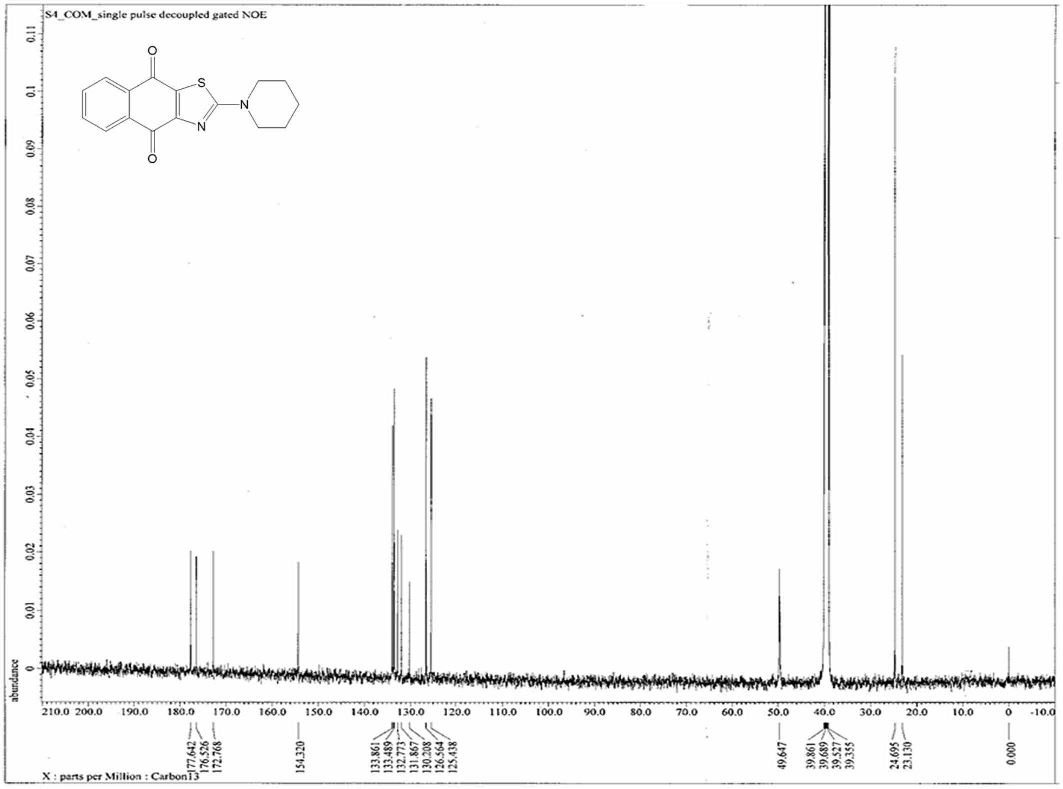

Figure S16. ^{13}C NMR spectrum (125 MHz, DMSO-d$_6$) of **5d**

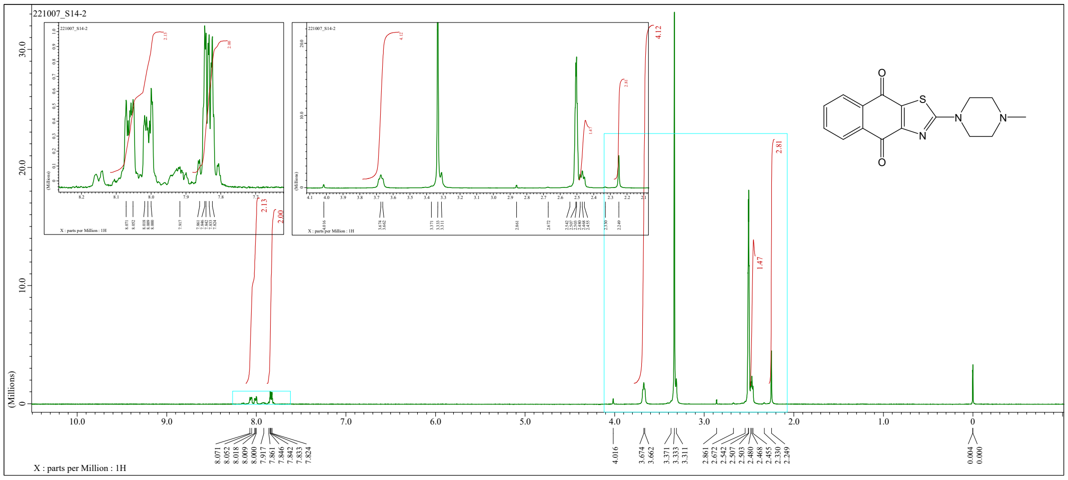

Figure S17. ^{1}H NMR spectrum (400 MHz, DMSO-d$_6$) of **5e**

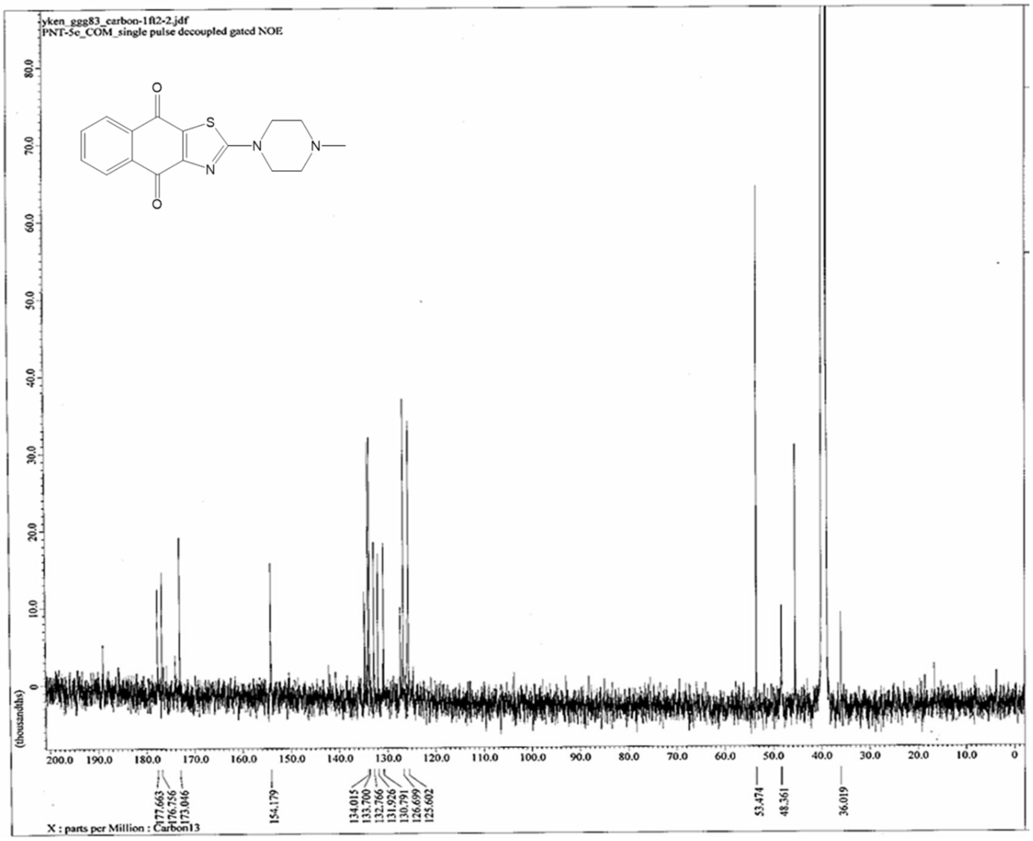

Figure S18. ^{13}C NMR spectrum (125 MHz, DMSO-d$_6$) of **5e**

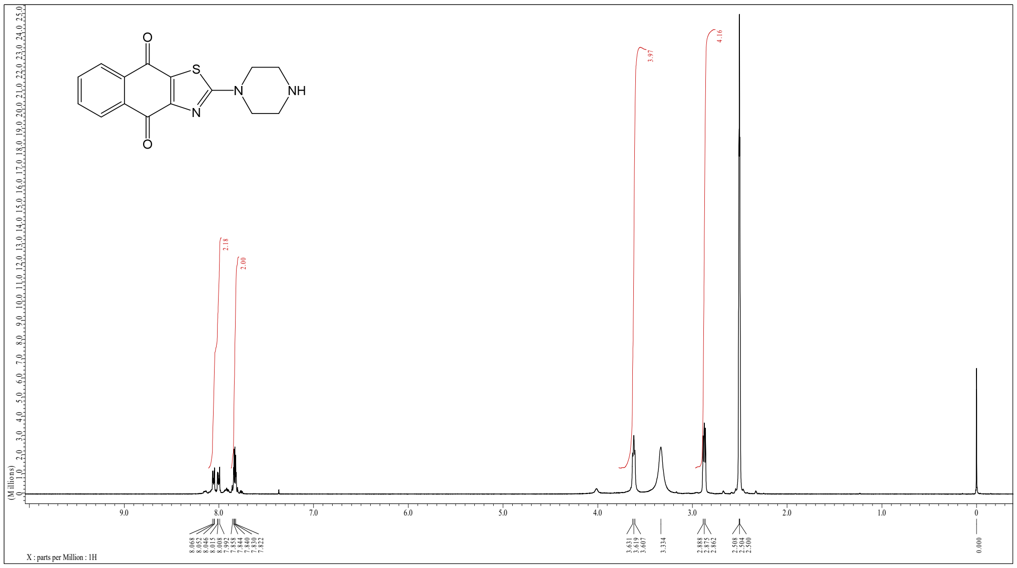

Figure S19. ^{1}H NMR spectrum (400 MHz, DMSO-d_6) of **PNT** [22]

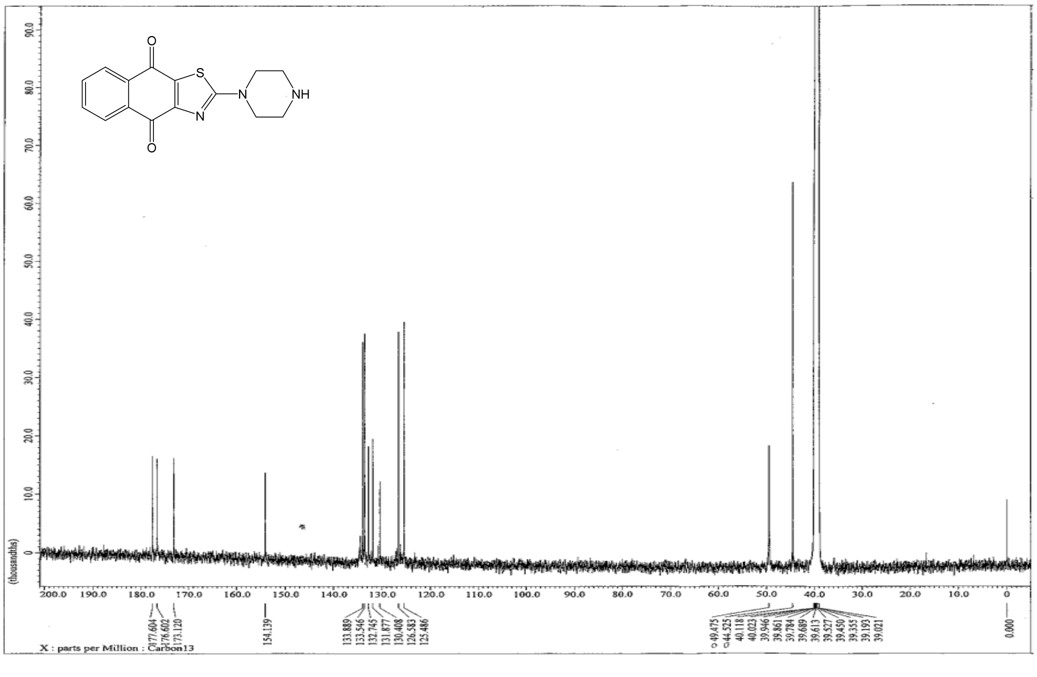

Figure S20. ^{13}C NMR spectrum (125 MHz, DMSO-d_6) of **PNT** [22]
